# Influence of on-scene time and prehospital interventions on inhospital mortality in trauma patients

**DOI:** 10.1186/s12873-025-01324-7

**Published:** 2025-08-20

**Authors:** Meng-Yu Wu, Giou-Teng Yiang, Sy-Jou Chen, Hon-Ping Ma, Mau-Roung Lin

**Affiliations:** 1 Department of Emergency Medicine, Taipei Tzu Chi Hospital, Buddhist Tzu Chi Medical Foundation, New Taipei, Taiwan, ROC; 2 School of Medicine, Tzu Chi University, Hualien, Taiwan, ROC; 3 Graduate Institute of Injury Prevention and Control, Taipei Medical University, 250 Wu-Hsing Street, Taipei, 11031 Taiwan, ROC; 4 Department of Emergency Medicine, Tri-Service General Hospital, National Defense Medical Center, Taipei, Taiwan, ROC; 5 Department of Emergency Medicine, Shuang Ho Hospital, Taipei Medical University, New Taipei City, Taiwan, ROC; 6 Department of Emergency Medicine, School of Medicine, Taipei Medical University, Taipei, Taiwan, ROC; 7 Programs in Medical Neuroscience, College of Medical Science and Technology, Taipei Medical University, Taipei, Taiwan, ROC

**Keywords:** Traumatic injury, On-scene time, Prehospital management, Prehospital transportation

## Abstract

**Objective:**

This study aimed to investigate the impact of the type and number of prehospital interventions, in addition to prehospital time intervals, on inhospital mortality among trauma patients.

**Methods:**

According to a 13-year prospective trauma registry, three prehospital time intervals of response time, on-scene time, and transport time were assessed. Prehospital interventions were classified into four categories: stop bleeding strategies, immobilization, basic airway management, and advanced resuscitation.

**Results:**

A total of 13,533 patients were included. Relative to patients not receiving prehospital interventions, there was longer on-scene time for those who received immobilization (10.4 vs. 8.68 min), basic airway management (11.4 vs. 9.40 min), and advanced resuscitation (12.6 vs. 9.53 min). Furthermore, relative to patients who survived in hospital, those who died significantly had longer on-scene time (11.4 vs. 9.64 min) and sustained immobilization (74.7% vs. 52.5%), basic airway management (30.7% vs. 7.6%), advanced resuscitation (32.0% vs. 0.1%), and ≥ 4 prehospital interventions (30.4% vs. 4.6%). Results of the multivariable logistic regression analysis shows that without adjustment for type and number of prehospital interventions, longer on-scene time (odds ratio [OR] = 1.03; 95% confidence interval [CI], 1.01–1.04) were significantly associated with increased mortality; however, after additional adjustment for type and number of prehospital interventions, no significant association between each of the three prehospital time intervals and mortality was detected. Furthermore, compared to patients who did not receive prehospital interventions, those who received wound packing/compression had a significantly lower risk of mortality (OR = 0.54; 95% CI, 0.36–0.80), whereas those who received basic airway management (OR = 1.73; 95% CI, 1.15–2.60), advanced resuscitation (OR = 33.4; 95% CI, 14.9–75.0), and three (OR = 2.60; 95% CI, 1.01–6.93) and ≥ 4 (OR = 2.97; 95% CI, 1.01–9.63) prehospital interventions had a significantly higher risk of mortality.

**Conclusions:**

There exists a tradeoff between prehospital trauma interventions and shortening on-scene time for mortality risk; however, implementing some interventions for hemorrhage control and hemostatic resuscitation can benefit the survival of trauma patients the most.

**Clinical trial number:**

Not applicable.

**Supplementary Information:**

The online version contains supplementary material available at 10.1186/s12873-025-01324-7.

## Introduction

Time to definitive care is a critical factor that can significantly impact patient outcomes [1]. The initial post-injury period within the first hour is traditionally perceived as the “golden hour”, emphasizing the crucial importance of prompt stabilization and rapid transfer to optimize patient outcomes by minimizing prehospital time [2–5]. The “load-and-go” approach has been proposed to shorten prehospital time for early transfer to the hospital for definitive intervention [6]. Conversely, the “stay-and-play” approach emphasizes the broad dissemination of trauma care training and the common use of hemostatic agents and blood transfusions, leading to a growth trend of advanced primary treatment and stabilization in the prehospital setting [7]. While there are no consensus on the influence of prehospital time on mortality risk in trauma populations [8–13], the debate regarding taking the “load-and-go” or “stay-and-play” for trauma patients still remains. This inconsistence might result from variations in the emergency medical service (EMS) systems, prehospital interventions, injury patterns, and environment factors [14–17]. Although prehospital interventions may impact the length of prehospital time, making their relationships be complex, few studies have explored both prehospital interventions and prehospital time on trauma outcomes.

Prehospital interventions that can vary according to the region, EMS system, and trauma care provider usually include strategies to stop bleeding, immobilization of injured limbs, basic airway management, and advanced resuscitation. These interventions at prehospital are selected based on their alignment with the Airway, Breathing, Circulation, Disability, and Exposure (ABCDE) approach [18]. The organization and operation for different types of prehospital interventions require varying duration of time, depending on the complexity of their technical execution. For instance, advanced airway management, such as endotracheal intubation, requires more on-scene time compared to the use of tourniquets [19]. Moreover, relative to basic prehospital care, advanced resuscitation may not only increase the on-scene time but also involve more prehospital interventions, while the number of interventions received at prehospital also depends on injury severity and injury progression, which are also associated with an increased prehospital time [20]. However, few have examined the impact of the type and number of prehospital interventions on trauma outcomes within the framework of prehospital time, although several studies have investigated the association between prehospital time and trauma outcomes.

Prior studies did not find a significant association between prolonged total prehospital time and mortality [21, 22]. A study based on the multicenter, international trauma registry also showed no significant associations of longer intervals of scene-to-hospital time and total prehospital time with the risk of 30-day mortality, although the two time intervals were significantly associated with poorer functional outcome [23]. The use of total prehospital time or scene-to-hospital time might dilute the effectiveness of specific prehospital time (e.g., on-scene time). The influence of response time, on-scene time, and transport time on trauma outcomes have been investigated but the results are inconsistent across studies [24–26].

Therefore, this study aimed to determine the impacts of the type and number of prehospital interventions and three prehospital time intervals of response time, on-scene time, and transport time on inhospital mortality among trauma patients.

## Methods

### Study design and setting

This study was a cohort study of patients presenting to the emergency department (ED) from the Tzu Chi trauma database and received approval from the Institutional Review Board of Taipei Tzu Chi Hospital in Taiwan. This trauma registry prospectively includes patients from four Tzu Chi hospitals, located in Taipei, Taichung, Dalin, and Hualien, respectively. The four hospitals are a medical center or a regional teaching hospital, and each of them has a capacity of at least 923 beds and 55 intensive care unit (ICU) beds. The study reported results adhered to the Strengthening the Reporting of Observational Studies in Epidemiology (STROBE) guidelines [27].

### Data source and study population

The Tzu Chi trauma registry is created due to the accreditation of emergency capacity on quality of care. For the trauma registry, registered case managers must undergo certification and continuing training courses provided by the Formosa Association for the Surgery of Trauma [28, 29], and the quality of registered data is reviewed monthly. The database consists of trauma patients with ICD-9-CM codes 800–959 (excluding those 905–909 and 930–939) or ICD-10-CM codes S00-T98 (excluding those T15-T19 and T90-T98) who required hospitalization.

For this study, we examined adult patients aged ≥ 20 years who presented to any of the four Tzu Chi hospitals between January 1, 2009 to December 31, 2021, with time-sensitive injuries (e.g., traffic road collisions, polytrauma, traumatic brain injuries, and falls). On the contrary, patients who had no information on prehospital time, were transferred from other hospitals or transported by helicopter, or were not transported by EMS systems were excluded from this study. Variables included in this study consisted of demographics, injury characteristics, prehospital time intervals, type and number of prehospital interventions, and inhospital mortality.

### Prehospital time measures

This study assessed three prehospital time intervals, including response time (from the time of injury to the time of EMS arrival on scene), on-scene time (from the time of EMS arrival on scene to the time of leaving the scene), and transport time (from the time of leaving the scene to the time of EMS arrival at the ED). After calculating the three prehospital time intervals, those data lower than 0 min were treated as missing value. Because the average response time was 4.1–4.9 min in urban areas and 6.6 min in rural areas based on previous studies [30], response time higher than 60 min was considered unreasonable data and potential outliers. These missing data and outliers were excluded for analysis.

### Prehospital interventions

Prehospital interventions were classified into four categories: stop bleeding strategies, immobilization, basic airway management, and advanced resuscitation. Stop bleeding strategies included external direct compression and tourniquet use. Immobilization procedures and techniques consisted of the use of neck collar for cervical-spine immobilization, long/short backboards, the Kendrick extrication device for thoracolumbar immobilization, and splints and triangular bandages for limbs. Basic airway management consisted of suction for blood or discharge, use of a nasal cannula (oxygenation support: 1–6 L per minute), and a simple mask (oxygenation support: 6–10 L per minute). Advanced resuscitation included advanced airway management of laryngeal mask airway and endotracheal intubation, cardiopulmonary resuscitation, and use of an external defibrillator.

The number of prehospital interventions was calculated as the sum of each intervention. For examples, when a patient received external direct compression, cervical-spine immobilization, suction for blood or discharge, and a simple mask used, four prehospital interventions were counted; when a patient received cervical-spine immobilization, long-backboard immobilization, and splints immobilization for injured limbs, three prehospital interventions were counted.

### Covariates

Covariates included demographics (age and sex), hospital area (rural or urban), years of (2009–2015 or 2016–2021), injury severity score (ISS), severe traumatic brain injury, injury type, and injury mechanism.

The ISS is calculated by summing the squares of the three highest Abbreviated Injury Scale (AIS) scores; higher ISS scores are indicative of more severe anatomical injuries [31, 32]; ISS scores were classified into four severity levels: <9, 9 ~ 15, 16 ~ 24, and ≥ 25. Severe traumatic brain injury was indicated by head AIS ≥ 3 while head AIS scores < 3 indicated absence of severe traumatic brain injury. The type of injury included penetrating (cutting, stab wounds, gunshot, or injuries caused by sharp objects like knives) and blunt (contusion or crushing) trauma. The mechanism of injury included road traffic collisions, falls, and others (violence, burns, cold injury, drowning, and electrical shocks).

### Inhospital mortality

The inhospital mortality referred to trauma deaths that occurred during the acute phase of treatment within the four Tzu Chi hospitals before discharge, including patients who died in the ED, ICU, or general wards [33].

### Statistical analysis

The normal distribution of continuous variables was assessed using the Kolmogorov-Smirnov test. Continuous variables exhibiting non-normal distribution were presented as medians with interquartile ranges (IQR) and analyzed using non-parametric analysis of variance or Mann–Whitney U tests. For continuous variables with normal distribution, the mean with standard deviation (SD) and independent T-tests were employed. For categorical variables, numbers and percentages were reported and Pearson’s chi-squared or Fisher’s exact tests were applied. The binary logistic regression analysis was used to determine the independent associations of prehospital time measures and prehospital interventions with inhospital mortality in trauma patients. Variables with *p* < 0.10 on the Pearson’s chi-squared or the Mann–Whitney U test were selected for multivariable analyses using the forced entry method. Variance inflation factors (VIFs) were utilized to identify multicollinearity among explanatory variables, and those variables with VIFs exceeding 10 were excluded from the analysis.

The complete case analysis was implemented under the assumption of missing at random mechanism [34], in which missing data on prehospital time intervals and clinical outcomes were excluded while those on age, sex, or other physiological variables were included. On the other hand, performing single or multiple imputation on missing data could potentially result in inaccurate estimations of prehospital time measures because the duration of prehospital time can be influenced by diverse factors, such as the distance between the injury scene and the hospital, city regulations, and/or EMS dispatching decisions and the information on these factors had not been collected in our registries. Furthermore, a comparison of patient characteristics between individuals included in the analysis and those with missing data on prehospital time intervals or prehospital intervention and inhospital mortality was conducted. All statistical tests were 2-sided and the significance level was set at 0.05. All the data analyses were performed using SPSS version 25.0 for Windows (IBM, Armonk, NY, USA).

## Results

**Fig. 1:**
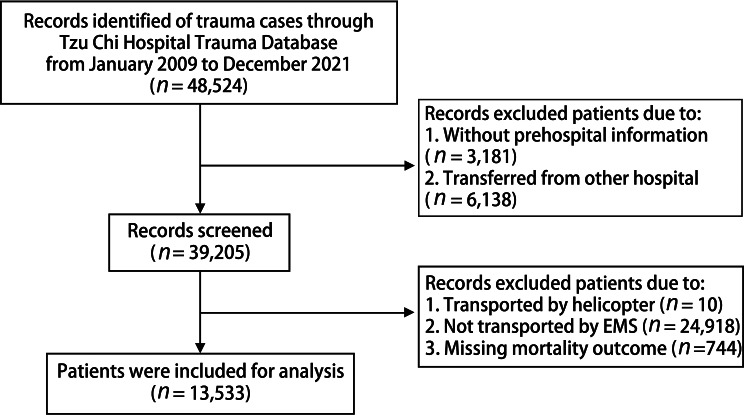
Flow diagram of study participants

Of 48,524 patients were registered in the Tzu Chi trauma database, 13,533 patients were included in this study and 34,991 patients were excluded due to the absence of prehospital information (*n* = 3,181), transfers from another hospital (*n* = 6,138), transportation by helicopter (*n* = 10), not transported by EMS (*n* = 24,918), and missing information on mortality (*n* = 744). The flow diagram of study participants is illustrated in Fig. 1.

The distributions of the prehospital time measures and prehospital interventions between patients who survived and those who died in hospital are presented in Table 1. The mean of response time, on-scene time, and transport time, respectively, were 8.69, 9.74, and 7.98 min. Compared to patients who survived in hospital, those who died were more likely to have longer intervals of on-scene time (11.4 vs. 9.64 min), while no significant differences in the response time and transport time between the two groups were found. Also, patients who died in hospital were more likely to be older, males, and injured in years of 2016–2021, to have ISS ≥ 25, severe traumatic brain injury, penetrating injuries, violence-related injuries and others, and to receive immobilization, basic airway management, advanced resuscitation, and a higher number of prehospital interventions, compared to those who survived in hospital.

**Table 1:** Comparisons of prehospital time measures and other characteristics between patients who survived and those who died in hospital

Characteristics	Total (*N* = 13533)	Survivors (*N* = 12757)	Deaths (*N* = 776)	*p*-value
Prehospital time, mean ± SD				
Response time	8.69 ± 7.27	8.69 ± 7.25	8.73 ± 7.72	0.907
On-scene time	9.74 ± 6.82	9.64 ± 6.52	11.4 ± 10.4	< 0.001
Transport time	7.98 ± 7.92	7.99 ± 7.97	7.80 ± 7.07	0.523
Age (years), median (IQR)	58(35–74)	57(35–74)	64(44–79)	< 0.001
20 ≤ age < 40	3803(28.1%)	3641(28.5%)	162(20.9%)	< 0.001
40 ≤ age < 60	3290(24.3%)	3108(24.4%)	182(23.5%)	
60 ≤ age < 80	4060(30.0%)	3815(29.9%)	245(31.6%)	
age ≥ 80	2378(17.6%)	2191(17.2%)	187(24.1%)	
Sex, n (%)				< 0.001
Female	6070(44.9%)	5813(45.6%)	252(32.6%)	
Male	7461(55.1%)	6939(54.4%)	522(67.4%)	
Years of injury				< 0.001
2009–2015	6199(45.8%)	5928(46.5%)	271(34.9%)	
2016–2021	7334(54.2%)	6829(53.5%)	505(65.1%)	
Hospital area				0.392
Rural	6618(48.9%)	6251(49.0%)	367(47.4%)	
Urban	6913(51.1%)	6506(51.0%)	407(52.6%)	
Injury severity score (ISS)				
ISS, mean ± SD	10.6 ± 11.2	8.92 ± 6.62	39.8 ± 25.5	< 0.001
ISS < 9	5207(38.5%)	5141(40.3%)	66(8.5%)	< 0.001
9 ≤ ISS < 16	5841(43.2%)	5756(45.1%)	85(11.0%)	
16 ≤ ISS < 25	1410(10.5%)	1311(10.3%)	99(11.8%)	
ISS ≥ 25	1075(8.0%)	549(4.3%)	526(67.8%)	
Severe traumatic brain injury				< 0.001
No	10,349(76.9%)	10,185(80.0%)	164(22.6%)	
Yes	3113(23.1%)	2550(20.0%)	563(77.4%)	
Injury type				< 0.001
Penetrating injury	361(2.7%)	346(2.7%)	15(1.9%)	
Blunt injury	13,172(97.3%)	12,411(97.3%)	761(98.1%)	
Mechanism of injury				< 0.001
Road traffic collisions	6790(50.2%)	6458(50.6%)	332(42.9%)	
Falls	4107(30.3%)	3867(30.3%)	240(30.9%)	
Others (violence, burns, cold, drowning, & electrical shock)	2634(19.5%)	2432(19.1%)	202(26.1%)	
Prehospital interventions				
No prehospital intervention	2333(19.7%)	2269(20.3%)	64(9.5%)	< 0.001
Basic traumatic life support	9258(78.3%)	8865(79.5%)	393(58.5%)	< 0.001
Wound packing/compression	6407(54.2%)	6089(54.6%)	318(47.3%)	< 0.001
Immobilization	6353(53.7%)	5851(52.5%)	502(74.7%)	< 0.001
Basic airway management	1058(8.9%)	852(7.6%)	206(30.7%)	< 0.001
Advanced traumatic life support	231(2.0%)	16(0.1%)	215(32.0%)	< 0.001
Number of prehospital interventions				< 0.001
0	2333(19.7%)	2269(20.3%)	64(9.5%)	
1	4658(39.4%)	4552(40.8%)	106(15.8%)	
2	2493(21.1%)	2385(21.4%)	108(16.1%)	
3	1621(13.7%)	1431(12.8%)	190(28.3%)	
≥4	717(6.1%)	513(4.6%)	204(30.4%)	

The distributions of prehospital time measures with regard to patient characteristics and prehospital interventions are presented in Table 2. Longer response time was significantly associated with the age group of ≥ 80 years, male sex, rural area, years of 2009–2015, ISS ≥ 25, non-severe-traumatic-brain-injury, absence of wound packing/compression, and a higher number of interventions. Longer on-scene time was significantly associated with the age group of ≥ 80 years, urban area, years of 2016–2021, ISS ≥ 25, penetrating injuries, falls, immobilization, basic airway management, advanced resuscitation, and a higher number of interventions. Longer transport time was significantly associated with male sex, rural area, years of 2016–2021, higher ISS scores, falls, immobilization, basic airway management, and a higher number of interventions.

**Table 2:** Comparisons of three prehospital time intervals in subgroups with regard to selected variables

Subgroups	Response time mean ± SD	On-scene time mean ± SD	Transport time mean ± SD
Age (years)			
20 ≤ age < 40	8.67 ± 6.75*	9.47 ± 6.74*	8.24 ± 9.00
40 ≤ age < 60	8.77 ± 7.26*	9.76 ± 7.61*	8.40 ± 9.14
60 ≤ age < 80	8.56 ± 7.19*	9.55 ± 6.79*	7.64 ± 6.64
age ≥ 80	8.87 ± 8.32*	10.5 ± 5.66*	7.58 ± 5.99
Sex			
Female	8.47 ± 7.24*	9.73 ± 6.07	7.62 ± 7.23*
Male	8.87 ± 7.29*	9.75 ± 7.37	8.28 ± 8.42*
Hospital area			
Rural	8.92 ± 6.67*	8.97 ± 6.29*	8.52 ± 8.93*
Urban	8.49 ± 7.78*	10.5 ± 7.21*	7.47 ± 6.78*
Years of injury			
2009–2015	9.04 ± 7.73*	9.16 ± 5.93*	7.65 ± 7.56*
2016–2021	8.41 ± 6.87*	10.2 ± 7.45*	8.26 ± 8.20*
Injury severity score (ISS)			
ISS < 9	8.53 ± 6.78*	8.92 ± 6.45*	7.75 ± 7.67*
9 ≤ ISS < 16	8.86 ± 7.54*	10.3 ± 6.12*	8.07 ± 7.97*
16 ≤ ISS < 25	8.43 ± 7.54*	9.82 ± 7.95*	8.16 ± 8.13*
ISS ≥ 25	8.99 ± 7.82*	10.7 ± 9.57*	8.38 ± 8.49*
Severe traumatic brain injury			
No	8.81 ± 7.42*	9.78 ± 6.82	8.01 ± 7.81
Yes	8.27 ± 6.77*	9.60 ± 6.77	7.87 ± 8.27
Injury type			
Blunt injury	8.67 ± 7.26	9.80 ± 6.81*	7.98 ± 7.92
Penetrating injury	9.41 ± 7.56	7.78 ± 6.57*	7.96 ± 8.00
Injury mechanism			
Road traffic collisions	8.46 ± 6.40*	8.90 ± 5.46*	7.75 ± 8.37*
Falls	9.67 ± 8.40*	10.8 ± 8.00*	8.38 ± 7.86*
Others (violence, burns, cold, drowning & electrical shock)	7.97 ± 7.59*	10.2 ± 7.64*	7.97 ± 6.71*
Prehospital interventions			
Wound packing/compression			
No	9.09 ± 7.72*	9.99 ± 6.92*	7.91 ± 7.98
Yes	8.67 ± 6.81*	9.25 ± 6.58*	8.16 ± 8.39
Immobilization management			
No	8.86 ± 7.36	8.68 ± 6.09*	7.60 ± 7.26*
Yes	8.85 ± 7.11	10.4 ± 7.17*	8.43 ± 8.93*
Basic airway management			
No	8.87 ± 7.26	9.40 ± 6.31*	7.94 ± 8.13*
Yes	8.71 ± 6.80	11.4 ± 9.78*	9.05 ± 8.84*
Advanced traumatic life support			
No	8.85 ± 7.21	9.53 ± 6.60*	8.05 ± 8.24
Yes	9.14 ± 7.90	12.6 ± 11.6*	7.75 ± 6.42
Number of prehospital interventions			
0	8.94 ± 7.93*	9.14 ± 6.11*	7.44 ± 7.08*
1	9.03 ± 7.26*	8.89 ± 6.12*	7.89 ± 7.70*
2	8.82 ± 7.05*	9.91 ± 6.34*	8.30 ± 9.31*
3	8.22 ± 6.50*	10.4 ± 7.50*	8.30 ± 8.35*
≥ 4	9.03 ± 6.93*	12.6 ± 10.2*	9.54 ± 9.98*

*P < 0.05, comparisons of the five prehospital time measures between each subgroup

**Fig. 2:**
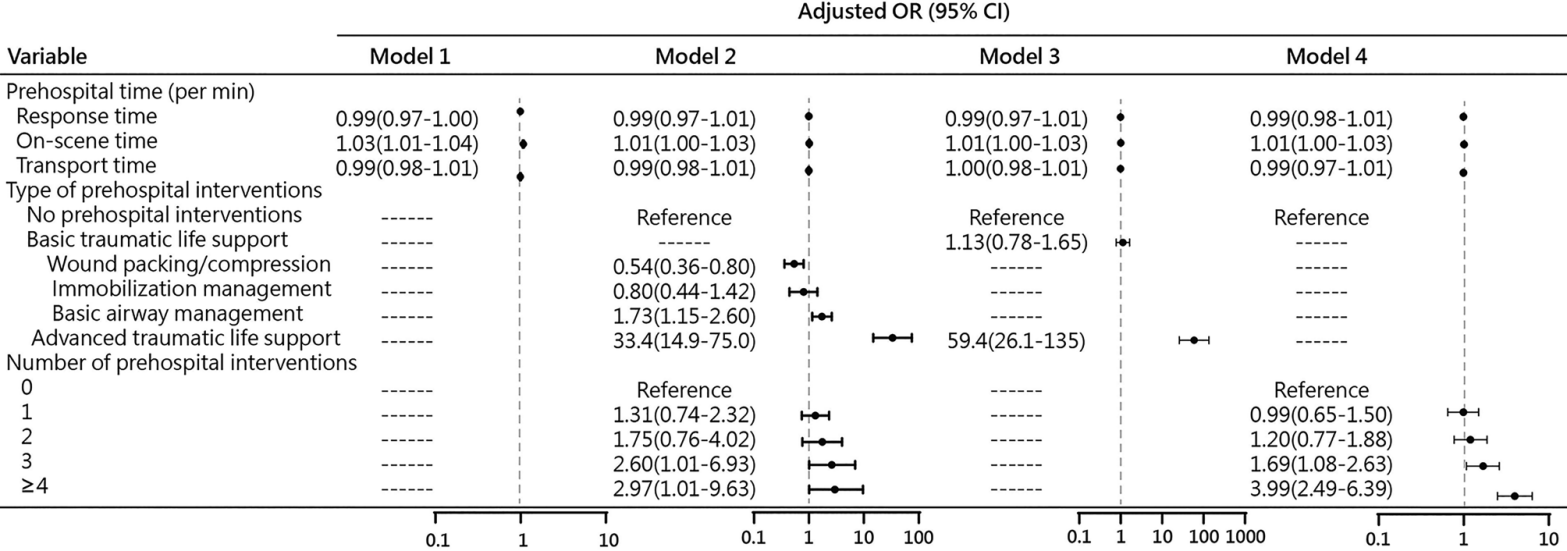
Forest plot of multivariable logistic regression models 1–4 for in-hospital mortality among trauma patients, adjusted for age, sex, year of injury, hospital area, Injury Severity Score (ISS), severe traumatic brain injury (TBI), injury type, and mechanism of injury. Adjusted odds ratios (OR) are shown with 95% confidence intervals (CI) on a logarithmic scale, with the vertical line at OR = 1 indicating no association

Results of the multivariable logistic regression model for inhospital mortality are shown in Table 3; Fig. 2. After adjustment for age, sex, ISS scores, hospital area, severe traumatic brain injury, injury type, injury year, and injury mechanism, longer on-scene time (odds ratio [OR] = 1.03; 95% confidence interval [CI], 1.01–1.04) was significantly associated with increased mortality while the response time and transport time were not (Model 1). Nevertheless, after additional adjustment for type and number of prehospital interventions, the association of on-scene time with mortality became non-significant (OR = 1.01; 95% CI, 1.00-1.03) (Model 2); furthermore, compared to patients who did not receive prehospital interventions, those who received wound packing/compression (OR = 0.54; 95% CI, 0.36–0.80) were significantly less likely to have died in hospital, while those who received basic airway management (OR = 1.73; 95% CI, 1.15–2.60), advanced resuscitation (OR = 33.4; 95% CI, 14.9–75.0), and three (OR = 2.60; 95% CI, 1.01–6.93) and ≥ 4 (OR = 2.97; 95% CI, 1.01–9.63) prehospital interventions were significantly more likely to have died in hospital.

**Table 3:** Multivariable logistic regression for inhospital mortality among trauma patients

Variable	Adjusted OR (95% CI)	
	Model 1	Model 2
Prehospital time (per min)		
Response time	0.99(0.97-1.00)	0.99(0.97–1.01)
On-scene time	1.03(1.01–1.04)	1.01(1.00-1.03)
Transport time	0.99(0.98–1.01)	0.99(0.98–1.01)
Age (years)		
20 ≤ age < 40	Reference	Reference
40 ≤ age < 60	1.19(0.87–1.62)	1.30(0.88–1.92)
60 ≤ age < 80	1.58(1.17–2.12)	2.23(1.55–3.22)
age ≥ 80	3.53(2.50–4.98)	5.68(3.72–8.68)
Sex		
Female	Reference	Reference
Male	1.18(0.94–1.46)	1.35(1.04–1.74)
Injury severity score (ISS)		
ISS < 9	Reference	Reference
9 ≤ ISS < 16	2.47(1.41–4.33)	2.56(1.36–4.81)
16 ≤ ISS < 25	9.54(5.25–17.4)	10.4(5.33–20.5)
ISS ≥ 25	149(84.8–263)	92.3(47.9–178)
Injury year		
2009–2015	Reference	Reference
2016–2021	1.00(0.80–1.25)	1.09(0.85–1.39)
Severe traumatic brain injury		
No	Reference	Reference
Yes	2.09(1.59–2.76)	1.86(1.34–2.59)
Injury type		
Blunt injury	Reference	Reference
Penetrating injury	2.42(1.05–5.62)	3.06(1.16–8.08)
Injury mechanism		
Road traffic collisions	Reference	Reference
Fall	1.80(1.38–2.34)	1.69(1.25–2.29)
Others (violence, burns, cold, drowning & electrical shock)	1.82(1.36–2.42)	1.68(1.07–2.64)
Type of prehospital interventions		
No prehospital interventions	------	Reference
Basic traumatic life support	------	
Wound packing/compression	------	0.54(0.36–0.80)
Immobilization management	------	0.80(0.44–1.42)
Basic airway management	------	1.73(1.15–2.60)
Advanced traumatic life support	------	33.4(14.9–75.0)
Number of prehospital interventions		
0	------	Reference
1	------	1.31(0.74–2.32)
2	------	1.75(0.76–4.02)
3	------	2.60(1.01–6.93)
≥ 4	------	2.97(1.01–9.63)

After separately adjustment for patient characteristics and type of prehospital interventions or number of prehospital interventions, on-scene time intervals were not significantly associated with mortality (Fig. 2 and Supplement Table 1). Among missing data, 16.6% were from response time, while any of other variables accounted for < 0.3%. Compared patients with complete information, those with missing data were more likely to be older, females, and injured in years of 2005–2015 and rural areas, as well as to have severe traumatic brain injury, falls, and longer intervals of on-scene time and transport time, but the mortality rate was not significantly different between the two groups (Supplement Table 2).

## Discussion

Our results showed that on-scene time was significantly associated with increased risk of inhospital mortality only when prehospital interventions were not considered; however, after accounting for type and number of prehospital interventions, all the three prehospital time measures of response time, on-scene time, and transport time were not significantly associated with mortality risk. In addition, compared to patients not receiving prehospital interventions, those who received basic airway management, advanced resuscitation, and three or more prehospital interventions were at increased risk of mortality, whereas those receiving wound packing/compression had a reduced risk of mortality.

In this study, both the type and number of prehospital interventions were significantly associated with on-scene time and mortality risk, and after adjusting for type and number of prehospital interventions, the apparent association between on-scene time and mortality was significantly weakened. Many studies have reported that shorter on-scene time reduced the risk of mortality [14, 35]. For instance, a 14-year trauma registry study revealed an increased odds of inhospital mortality among patients with penetrating injury, in which those with on-scene time > 20 min vs. on-scene time < 10 min had an OR of 2.90 for increased mortality [10]. However, these studies did not consider the moderating effect of prehospital interventions on the relationship between on-scene time and mortality risk. After considering prehospital interventions, such as intravenous or intraosseous line placed, hemorrhage control, tracheal intubation attempt, and rescue airway, like our result, the association between on-scene time and mortality no longer existed [12, 36]. As inconsistent with our finding, some studies found a significant association of shorter response time or shorter transport time with mortality [23–26]. The inconsistence could be caused by the differences in EMS dispatch and personnel scheduling, geographical factors, mode of transportation (air or ground transport), and accessibility of trauma center [20].

Consistent with our findings, previous studies have also demonstrated that trauma patients receiving advanced prehospital interventions tend to have poorer outcomes. Specifically, each additional prehospital procedure was associated with approximately a 2.5-fold increase in the risk of in-hospital mortality [37] and advanced resuscitation did not improve survival of trauma patients [38], although these patients were already critically injured and near death at the time of intervention. However, whether a patient to receive basic or advanced interventions depends on the severity and type of sustained injuries, and those patients who receive advanced prehospital care, particularly when airway management is required, should sustain more-severe injuries, have greater complexity, and be at a higher risk of mortality, compared to those receiving basic prehospital care [39, 40]. In a previous study examining whether advanced airway interventions—such as prehospital endotracheal intubation and chest tube placement—were unnecessarily time-consuming in severely injured patients, the overall trauma resuscitation time, defined as the interval from the time of injury to the completion of initial emergency department management, was found to be similar regardless of whether the interventions were performed prehospital or in-hospital [41]. In all cases, the overall trauma resuscitation time (approximately 150 min) substantially exceeded the “golden hour.” Compared to patients who did not receive advanced airway interventions in the prehospital setting, those who did had poorer vital signs at the scene, sustained more severe injuries, and therefore received invasive procedures more frequently. However, after adjusting for trauma severity using the Trauma Score and Injury Severity Score (TRISS), there was no significant difference in either 24-hour or in-hospital mortality between the two groups. These findings suggest that patients who received advanced resuscitation were likely more critically injured to begin with. For severely injured patients, appropriate interventions should be administered as early as possible in the prehospital phase, rather than being delayed until arrival at the hospital. An analysis of prehospital and early inhospital trauma deaths also revealed that 73% of deaths were deemed anatomically non-survivable, 14% were anatomically survivable with advanced prehospital care, and only 4% were anatomically survivable with basic prehospital care [42]. On the contrary, patients who underwent wound packing/compression had a lower risk of mortality than those not receiving prehospital interventions, indicating that hemorrhage control and hemostatic resuscitation at prehospital are effective in reducing early mortality due to exsanguination as the leading cause of death in the prehospital setting [39]. This also reflects that external bleeding is generally easier to identify and manage, making hemorrhage control an appropriate and frequently life-saving intervention in most cases.

In this study, patients receiving ≥ 3 interventions at prehospital were at increased risk of mortality, compared to not receiving any intervention. Previous studies have also reported that patients those who died in the ED or were subsequently admitted to the ICU, relative to those who survived until hospital admission, received more prehospital interventions, and patients with > 2 prehospital interventions sustained lower survival than those with fewer interventions [43]. The number of interventions received at prehospital might depend not only on injury severity but also on the patient’s physical capacity and the dynamics of clinical deterioration [44]. Preexisting chronic diseases and medications might affect the body’s compensatory capacity after injury, leading to the need for more prehospital interventions to stabilize patients [45, 46]. For examples, patients with chronic pulmonary disease often have higher oxygen demands and higher risks for unplanned intubation during bleeding episodes [47–49], and cardiovascular patients may bring about misdiagnosis and additional interventions due to unobvious shock symptoms during bleeding [50–52]. Furthermore, due to the progression of traumatic injuries, patients with acute respiratory failure might necessitate additional interventions, such as endotracheal intubation and the use of supraglottic airway devices, to secure airways. Despite injury severity being controlled for in this study, some injury features (e.g., hemorrhagic shock), environmental factors, and emergency medical responder’s characteristics that could play a role on outcome were not considered in the result.

In addition, prehospital medical interventions should be determined and performed based on the specific injuries of each patient; a uniform or excessive application may have a negative impact on the outcomes of trauma patients, or at least fail to provide the anticipated benefits. In the study by Hussmann et al. [53], among severe TBI patients with concurrent hemorrhage, aggressive prehospital fluid therapy resulted in impaired coagulation and had no positive impact on outcomes, including mortality and length of stay (both in-hospital and ICU). Similarly, in TBI patients, even after adjusting for multiple confounding factors, prehospital intubation remained associated with poorer outcomes [54]. This may be attributable to prolonged on-scene time, technical issues related to intubation, and insufficient experience of paramedics. Therefore, the decision to perform prehospital intubation should be based on factors such as EMS resources, system structure, provider training level, the presence of an emergency physician at the scene, and transport time.

This study has some limitations. First, a substantial proportion of patients were excluded from analysis due to missing data on prehospital time intervals so that the study results might be susceptible to selection bias. Most of the patients excluded due to missing data lacked one of the prehospital time interval records of interest, rather than variables such as age, sex, or mechanism of injury. Therefore, we considered that it would be inappropriate to impute missing prehospital time intervals. In our study, missing data led to the exclusion of over 30% of the original cohort. Among the missing data, 16.6% were from response time. We fully acknowledge that differences in baseline characteristics between the included and excluded samples, as well as the missing data inherent to the retrospective design, may have introduced potential bias, particularly in the analysis of response time. In addition, we did not exclude patients who experienced out-of-hospital cardiac arrest in the prehospital phase. However, patients with obvious signs of death (e.g., traumatic decapitation) are not transported by EMS according to emergency protocols in our system and are thus not included in the trauma registry. To avoid selection bias—for example, excluding patients who were alive on-scene but experienced cardiac arrest en route to the hospital, or those who were in cardiac arrest at the scene and achieved return of spontaneous circulation during transport—we included all patients who were transported to the hospital in our analysis.

Second, we must acknowledge the possibility that evaluating both the impacts of prehospital interventions and prehospital time intervals on outcomes may not be feasible through an observational study alone. Some variables that could potentially affect mortality risk were not measured in this study, including experiences of emergency medical teams with prehospital interventions, extrication, communication with command or dispatch, the receiving center, bystander management of the patient, and quality of emergency medical teams. In addition, we lacked data on EMS provider training, years of experience, adherence to standard operating procedures, and scene logistics, all of which may have influenced intervention decisions and patient outcomes.

Third, we did not document the reasons for emergency medical teams choose to perform specific types or quantities of prehospital interventions for each patient. Situational factors, such as challenging extrication from the scene due to entrapment or navigating stairs, could vary case by case and contribute to prolonged on-scene times, especially in road traffic collisions. Furthermore, we did not exclude patients with prolonged prehospital times over 90–120 min. Although such cases were rare in our dataset (50 cases [0.36%] exceeded 90 min and only 15 [0.11%] exceeded 120 min), they were retained to reflect real-world EMS operational conditions. Notably, our trauma registry includes patients from large-scale disasters, such as the 2018 Taiwan Puyuma train derailment and the Hualien earthquake, where prolonged extrication and transport times are expected. However, since the trauma registry does not capture specific reasons for prolonged prehospital times, the potential impact of this variable on clinical outcomes could not be further analyzed and should be interpreted with caution. Fourth, the use of the ISS might be insufficient to the overall injury severity in trauma patients [55]. ISS scores can only account for the most severe injury in three body regions, potentially overlooking severe injuries in body regions other than the three and less severe injuries within the same region [56, 57]. Furthermore, the ISS only anatomical injuries but not account for physiological changes, such as hemorrhagic shock [58]. Finally, timely transfer of seriously injured patients to definitive care is a key indicator of trauma network performance, and the prehospital time is only a part of the time interval from the occurrence of an injury event to definitive care. The time to arrival at hospital may differ from the time to definitive care, such as the need for surgical or hemostatic interventions. The time to definitive care that can be influenced by hospital staff scheduling and the circumstances at the time might affect our result to demonstrate the association between prehospital time and mortality risk.

## Conclusions

This study demonstrated that longer on-scene time was significantly associated with increased risk of inhospital mortality only when prehospital interventions were not considered, and receiving basic airway management, advanced resuscitation, and three or more prehospital interventions was significantly associated with increased mortality, compared to those not receiving prehospital interventions. There exists a tradeoff between prehospital trauma interventions and shortening on-scene time for mortality risk; however, implementing interventions for hemorrhage control and hemostatic resuscitation can benefit the survival of trauma patients the most. However, it is important to recognize that the performance of interventions likely reflects the severity of injury and elevated mortality risk, rather than serving as a direct cause of death. This analysis is therefore not intended to determine whether prehospital interventions are beneficial or harmful, but rather describe their association with mortality within a real-world EMS context. Further studies employing more rigorous methodologies are warranted to validate and expand upon these findings.

## Supplementary Information

Below is the link to the electronic supplementary material.

Supplementary Material 1

## Data Availability

The data used or analyzed in this study are available from the corresponding author upon reasonable request.
