# Supplementary material for: Influence of on-scene time and prehospital interventions on inhospital mortality in trauma patients
Source: BMC Emerg Med. 2025 Aug 20;25:163. doi: 10.1186/s12873-025-01324-7 (PMC12366050; doi:10.1186/s12873-025-01324-7)
Supplement: Supplementary file 1 — Supplementary Material 1 [file 12873_2025_1324_MOESM1_ESM.docx]

**Supplement Table 1.** Multivariable logistic regression of separate adjustment of type and number prehospital interventions for inhospital mortality among trauma patients

| Variable | Adjusted OR (95% CI) | |
| --- | --- | --- |
|  | Model 3 | Model 4 |
| Prehospital time (per min) |  |  |
| Response time | 0.99(0.97-1.01) | 0.99(0.98-1.01) |
| On-scene time | 1.01(1.00-1.03) | 1.01(1.00-1.03) |
| Transport time | 1.00(0.98-1.01) | 0.99(0.97-1.01) |
| Age |  |  |
| 20 ≤ age < 40 | Reference | Reference |
| 40 ≤ age < 60 | 1.25(0.85-1.83) | 1.32(0.94-1.87) |
| 60 ≤ age < 80 | 1.95(1.36-2.78) | 1.98(1.42-2.75) |
| age ≥ 80 | 4.75(3.16-7.15) | 5.09(3.44-7.53) |
| Sex |  |  |
| Women | Reference | Reference |
| Men | 1.27(0.98-1.63) | 1.24(0.97-1.58) |
| Injury severity score (ISS) |  |  |
| ISS < 9 | Reference | Reference |
| 9 ≤ ISS < 16 | 2.85(1.53-5.32) | 2.66(1.43-4.96) |
| 16 ≤ ISS < 25 | 11.8(6.05-22.9) | 9.83(5.08-19.0) |
| ISS ≥ 25 | 124(65.2-234) | 119(62.9-226) |
| Years of injury |  |  |
| 2009-2015 | Reference | Reference |
| 2016-2021 | 1.04(0.81-1.32) | 1.05(0.83-1.33) |
| Traumatic brain injury |  |  |
| No | Reference | Reference |
| Yes | 1.77(1.28-2.45) | 1.95(1.44-2.65 |
| Injury type |  |  |
| Blunt injury | Reference | Reference |
| Penetrating injury | 2.26(0.87-5.85) | 2.02(0.83-4.89) |
| Injury mechanism |  |  |
| Road traffic collisions | Reference | Reference |
| Falls | 1.79(1.34-2.40) | 2.04(1.54-2.70) |
| Others (violence, burns, cold, drowning & electrical shock) | 1.79(1.15-2.77) | 2.88(1.96-4.25) |
| Type of prehospital interventions |  |  |
| No prehospital interventions | Reference | Reference |
| Basic traumatic life support | 1.13(0.78-1.65) | ------ |
| Wound packing/compression | ------ | ------ |
| Immobilization management | ------ | ------ |
| Basic airway management | ------ | ------ |
| Advanced traumatic life support | 59.4(26.1-135) | ------ |
| Number of prehospital interventions |  |  |
| 0 | ------ | Reference |
| 1 | ------ | 0.99(0.65-1.50) |
| 2 | ------ | 1.20(0.77-1.88) |
| 3 | ------ | 1.69(1.08-2.63) |
| ≥4 | ------ | 3.99(2.49-6.39) |

**Supplement Table 2.** Comparison of included and excluded patients due to missing prehospital times

| Characteristics | Missing data  n(%) | Included  patients |  | | Excluded Patients | |  |
| --- | --- | --- | --- | --- | --- | --- | --- |
|  |  |  | All excluded patients | p-value | | Prehospital times missing | p-value |
| Patient number, n | --- | 11118 | 34991 |  | | 2415 |  |
| Age (years) | 1024(7.5%) |  |  | <0.001 | |  | <0.001 |
| 20 ≤ age < 40 | --- | 2344(21.1%) | 6453(18.4%) |  | | 437(18.1%) |  |
| 40 ≤ age < 60 | --- | 2770(24.9%) | 9179(26.2%) |  | | 520(21.5%) |  |
| 60 ≤ age < 80 | --- | 3354(30.2%) | 11061(31.6%) |  | | 706(29.2%) |  |
| age ≥ 80 | --- | 1757(15.8%) | 5391(15.4%) |  | | 621(25.7%) |  |
| Sex, n (%) | 0(0.0%) |  |  | 0.104 | |  | 0.012 |
| Women | --- | 4931(44.4%) | 15982(45.7%) |  | | 1139(47.2%) |  |
| Men | --- | 6187(55.6%) | 19009(54.3%) |  | | 1276(52.8%) |  |
| Years of injury | 0(0.0%) |  |  | <0.001 | |  | <0.001 |
| 2009-2015 | --- | 4982(44.8%) | 9235(26.4%) |  | | 1217(50.4%) |  |
| 2016-2021 | --- | 6136(55.2%) | 23338(66.7%) |  | | 1198(49.6%) |  |
| Prehospital time, mean±SD |  |  |  |  | |  |  |
| Response time | 2252(16.6%) | 8.68±7.25 | 8.02±6.19 | 0.006 | | 9.66±8.66 | 0.151 |
| On-scene time | 64(0.5%) | 9.59±6.24 | 9.99±6.63 | 0.065 | | 10.5±9.01 | <0.001 |
| Transport time | 137(1.0%) | 7.78±7.25 | 9.38±11.1 | <0.001 | | 8.98±10.6 | <0.001 |
| Hospital area | 0(0.0%) |  |  | <0.001 | |  | <0.001 |
| Rural | --- | 5345(48.1%) | 25466(72.8%) |  | | 1273(52.7%) |  |
| Urban | --- | 5773(51.9%) | 9525(27.2%) |  | | 1142(47.3%) |  |
| Injury severity score (ISS) | 73(0.5%) |  |  | <0.001 | |  | <0.001 |
| ISS < 9 | --- | 4419(39.7%) | 19509(55.8%) |  | | 788(32.6%) |  |
| 9≤ISS < 16 | --- | 4631(41.7%) | 12111(34.6%) |  | | 1210(50.1%) |  |
| 16≤ ISS < 25 | --- | 1183(10.6%) | 2356(6.7%) |  | | 227(9.4%) |  |
| ISS ≥ 25 | --- | 885(8.0%) | 1015(2.9%) |  | | 190(7.9%) |  |
| 0Traumatic brain injury | 71 (0.5%) |  |  | <0.001 | |  | 0.014 |
| No | --- | 8460(76.5%) | 29930(85.5%) |  | | 1889(78.8%) |  |
| Yes | --- | 2605(23.5%) | 5061(14.5%) |  | | 508(21.2%) |  |
| Injury type | 0(0.0%) |  |  | <0.001 | |  | 0.618 |
| Blunt injury | --- | 10825(97.4%) | 32913(93.1%) |  | | 2347(97.2%) |  |
| Penetrating injury | --- | 293(2.6%) | 2078(6.9%) |  | | 68(2.8%) |  |
| Injury mechanism | 0(0.0%) |  |  | <0.001 | |  | <0.001 |
| Road traffic collisions | --- | 5780(52.0%) | 10576(35.5%) |  | | 1010(41.8%) |  |
| Falls | --- | 3084(27.8%) | 14597(41.7%) |  | | 1025(42.4%) |  |
| Others (violence, burns, cold, drowning &  electrical shock) | --- | 2254(20.3%) | 9818(28.1%) |  | | 380(15.7%) |  |
| Inhospital deaths | 0 (0.0%) | 633(5.7%) | 570(1.6%) | <0.001 | | 143(5.9%) | 0.662 |
